# Changes in variation at the MHC class II DQA locus during the final demise of the woolly mammoth

**DOI:** 10.1038/srep25274

**Published:** 2016-05-04

**Authors:** Patrícia Pečnerová, David Díez-del-Molino, Sergey Vartanyan, Love Dalén

**Affiliations:** 1 Department of Bioinformatics and Genetics, Swedish Museum of Natural History, SE-10405 Stockholm, Sweden; 2 Department of Zoology, Stockholm University, SE-10691 Stockholm, Sweden; 3 North-East Interdisciplinary Scientific Research Institute N.A.N.A. Shilo, Far East Branch, Russian Academy of Sciences (NEISRI FEB RAS), Magadan, Russia

## Abstract

According to the nearly-neutral theory of evolution, the relative strengths of selection and drift shift in favour of drift at small population sizes. Numerous studies have analysed the effect of bottlenecks and small population sizes on genetic diversity in the MHC, which plays a central role in pathogen recognition and immune defense and is thus considered a model example for the study of adaptive evolution. However, to understand changes in genetic diversity at loci under selection, it is necessary to compare the genetic diversity of a population before and after the bottleneck. In this study, we analyse three fragments of the MHC DQA gene in woolly mammoth samples radiocarbon dated to before and after a well-documented bottleneck that took place about ten thousand years ago. Our results indicate a decrease in observed heterozygosity and number of alleles, suggesting that genetic drift had an impact on the variation on MHC. Based on coalescent simulations, we found no evidence of balancing selection maintaining MHC diversity during the Holocene. However, strong trans-species polymorphism among mammoths and elephants points to historical effects of balancing selection on the woolly mammoth lineage.

Small populations face an increased risk of extinction due to loss of genetic diversity, which is associated with inbreeding depression and loss of adaptive variation^1^. Inbreeding can lead to unmasking of deleterious alleles, while a decreased adaptive potential affects the population’s ability to adapt to environmental changes or to respond to pathogens^2^.

In vertebrates, the capability to resist pathogens is determined by the variability of genes of the major histocompatibility complex (MHC)^3^. The MHC encodes proteins that are responsible for antigen recognition and initiation of an immune response. In order to recognize and bind diverse pathogens, the expressed loci of MHC are highly polymorphic and it is thought that this diversity is maintained by a pathogen-mediated balancing selection^4^,^5^. Among other factors, selection pressure and investment in immune defense are associated with environmental and climatic conditions^6^,^7^. It has been shown that pathogen and parasite loads are lower in high latitude, cold and arid environments and under increased solar radiation^8^,^9^,^10^.

What effect a bottleneck has on MHC diversity has been a long-standing topic of discussion in research on natural selection^11^. There are two basic scenarios: selection either maintains diversity throughout the bottleneck, or the diversity is lost due to the effects of genetic drift^12^. From a conservation perspective, understanding this is important since low MHC diversity is associated with decreased ability to resist pathogens^13^,^14^. Thus, populations with few MHC alleles are more susceptible to infectious diseases due to decreased heterozygosity^15^,^16^ or absence of a resistant allele^17^,^18^.

A meta-analysis of studies on the effect of bottlenecks on MHC polymorphism^12^ supports the latter scenario and shows that in most of the studied populations MHC diversity decreased as a consequence of the bottlenecks. However, there are examples where, in contrast to neutral diversity, MHC polymorphism appears to have been maintained during bottlenecks^19^,^20^, including endangered species with small population sizes^21^,^22^.

In recent years, however, analyses of DNA from historical samples have been applied to directly study pre- and post-bottleneck MHC diversity in prairie-chickens^23^, peary caribou^24^ and New Zealand passerines^25^. These studies suggest that balancing selection is not only unable to maintain MHC diversity throughout a bottleneck, but in prairie-chickens and passerines the decrease of variability in MHC loci was more profound than in neutral markers (i.e. microsatellites).

To date, no studies have examined changes in MHC diversity leading up to a species’ extinction. In this study, we have used ancient DNA from the woolly mammoth (*Mammuthus primigenius*) to study the change in diversity in a MHC locus as the species went through a bottleneck and subsequently persisted at a small population size until its extinction ~4 cal ka (thousand calendar years ago). The woolly mammoth was a large herbivore with a widespread Holarctic distribution during the Pleistocene, but retreated to the northern edge of its range following the climatic changes at the Pleistocene-Holocene boundary^26^. Wrangel Island was the terminal refugium where the woolly mammoth survived an additional six thousand years after the mainland populations went extinct^27^,^28^, with an estimated mean effective population size of 326 individuals^29^. Several studies, including a whole-genome analysis of one of the last surviving Wrangel mammoths, have shown that isolation on Wrangel Island led to a decline in genetic diversity by 20% in autosomal heterozygosity and ~37% in allelic richness, likely due to a combination of inbreeding and genetic drift in the population prior to its extinction^29^,^30^. While previous studies have identified declines in neutral genetic diversity prior to a species’ extinction^29^,^31^,^32^, there is a lack of knowledge on whether adaptive genetic diversity also declined prior to these extinctions. However, based on the meta-analysis discussed above, as well as previous studies on historical DNA in other taxa, we hypothesize that balancing selection was unable to maintain MHC diversity in the Wrangel population. We thus expect to find a similar loss in MHC diversity as that previously observed in neutral markers.

## Methods

### DNA extraction and primer design

**Figure 1:**
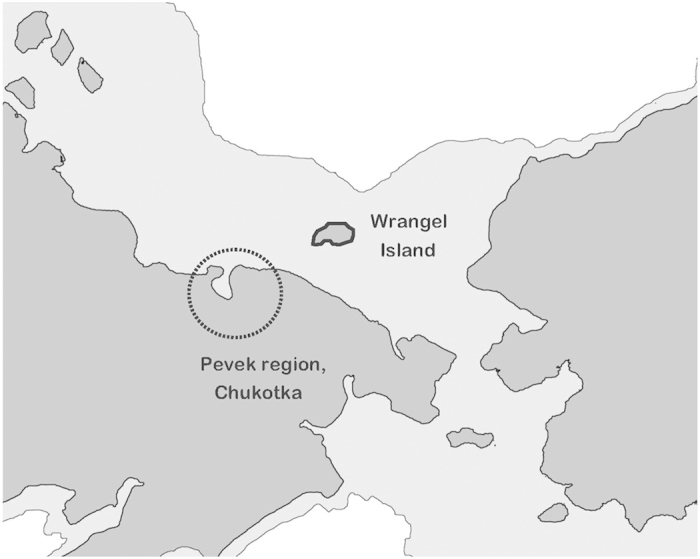
A map depicting the two sampling locations. Wrangel Island and Pevek Region, Chukotka are highlighted. The light shaded area depicts the extent of dry land in the Late Pleistocene, before Wrangel’s isolation^69^. The map was drawn using Adobe® Photoshop® software (CS5, version 12).

We analysed radiocarbon-dated samples (Table 1) representing *i*) the Pleistocene continental population in Chukotka and Wrangel while these areas were still a part of Beringia (>13 cal ka), and *ii*) the Wrangel Island population after rising sea levels led to its isolation (<10 cal ka; Fig. 1). We used a Dremel drill to obtain approximately 50 mg of powder from bones, teeth and tusks. DNA was extracted from the powder following a modified version^33^ of a silica-based ancient DNA extraction protocol^34^.

We designed four new primer pairs (see Supplementary Table S1) targeting four short fragments of the MHC DQA gene that have been shown to be variable in both Asian and African savannah elephants^35^. Two of these fragments were located in exons and two in introns. We tested the primers by amplifying and sequencing four mammoth samples with high levels of DNA preservation. Amplifications were performed using 1× PCR Buffer, 0.2 μM of each primer, 0.2 mM of each dNTP, 2.5 mM MgCl_2_, 0.1 mg/mL BSA, 2U (0.4 μL) HotStar Taq DNA Polymerase (Qiagen), 2 μL of DNA extract and distilled water up to the total volume of 25 μL. Amplifications were carried out under following conditions: 10 min of initial denaturation at 95 °C; 55 cycles of denaturation of 30 sec at 94 °C, annealing of 30 sec at 50–54 °C (depending on the primer pair, see Supplementary Table S1) and extension of 30 sec at 72 °C; followed by a final extension 7 min at 72 °C. PCR products were cleaned using Exo-FAP (Fermentas) and sequenced on an ABI 3130xl (Applied Biosystems). All primer pairs yielded sequences that were almost identical to elephant MHC sequences when blasted against the NCBI database^36^.

### 454 pyrosequencing

We screened 82 mammoth samples by amplifying a 141-bp fragment using primers MPDQA-1F and MPDQA-1R. Thirty-six samples yielded a single clear band of expected size and 32 of these were selected for subsequent analyses. To create a pooled library for sequencing on the 454 pyrosequencing platform, each primer was equipped with 8 unique six-nucleotide tags designed to differ at at least two positions so that the risk of incorrect sorting due to sequencing errors in the tag sequences was minimized^37^. As both forward and reverse primers were tagged, we were able to pool 64 samples per locus (256 in total). This allowed us to include two PCR replicates for each of the 32 selected samples on the same 454 sequencing run.

Amplifications with tagged primers were performed with the same concentrations and PCR conditions as described above; except for using 0.2 μM of each primer and using 5 μL of DNA extract instead of 2 μL. We were able to successfully amplify 181 of 256 PCR products and these were pooled and cleaned using the Qiagen MinElute PCR Purification Kit with elution volume of 20 μL. Three loci showed consistent results and were in the pool represented by 51 (exon 2) or 52 (both intron 2a and exon 4) PCR products. Intron 2b worked only for 26 samples, likely due to the fact that this fragment was longer than the other fragments that were targeted.

To estimate the concentration of the cleaned pool, we ran 2 μL of the pool on an agarose gel and compared it to a dilution series of a GeneRuler 100 bp Plus DNA Ladder (Fermentas). Subsequently, we followed a modified version^38^ of the Rapid Library Preparation Method Manual and Sequencing Method Manual supplied with the GS Junior System (454 Life Sciences). The pool was sequenced in one GS Junior run.

### Authenticity of results

We conducted all pre-PCR steps in a dedicated ancient DNA laboratory at the Swedish Museum of Natural History in Stockholm, which is physically separated from the modern DNA facilities. We used protective gloves, suits and face masks to prevent contamination. All surfaces were regularly cleaned with sodium hypochlorite and tools and reagents were cleaned by ultraviolet irradiation. We used negative controls with all extractions and PCRs (at least one per eight samples).

For samples that were scored as homozygotes, we added a third replicate to further reduce the risk that allelic dropout led to erroneous genotypes. These third replicates were sequenced by Sanger sequencing on the ABI 3130xl sequencer (Applied Biosystems) with primers MPDQA-1F, -1R and -3F being replaced by internal primers (see Supplementary Table S1) to secure a more efficient targeting of the mammoth MHC. We calculated the proportion of allelic dropout^39^ to verify that the probability of false negatives was lower than 0.05.

MHC is recognized as one of the hotspots of gene duplication in various taxonomic units^40^. However, there are three reasons why we are confident that our results represent variability at a single locus. First, a study of elephant MHC^35^ didn’t find any evidence of paralogues and showed that the DQA was successfully transcribed and expressed. Second, we never observed more than two alleles per locus in any of the mammoth individuals. Third, we mapped all alleles against the African elephant genome using the Burrows-Wheeler Aligner^41^ (BWA) and observed very high mapping quality scores (MP = 60), which strongly suggested that all alleles mapped only to a single position in the elephant genome.

### Data processing

We used the 454 Sequencing System Software 2.9 to process the sequencing data generated in the GS Junior run. Reads that passed system filters were sorted according to 5′ and 3′ tags using the sfffile command and allowing for one mismatch. The sorted files in .sff format were converted into fasta and quality files using the sffinfo command. We used a perl script fastaQual2fastq.pl (https://github.com/josephhughes/Sequence-manipulation/blob/master/fastaQual2fastq.pl) to convert the fasta and quality files into fastq files. At this point, each fastq file represented (one replicate of) one individual and contained reads for all loci that were successfully amplified for that particular sample.

The reads were mapped to the reference Asian elephant sequence (GenBank no. GU369701) following steps 4–6.6 of a mapping pipeline for 454 sequencing data^42^. BWA 0.7.10^41^ was used to index the reference sequence and mapped the reads against the indexed reference sequence with the bwa mem algorithm. We used SAMtools 1.1^43^ to convert the files containing mapped reads from SAM to BAM format, sort and index the BAM files. We performed the subsequent processing of BAM files in Geneious R7^44^. Primer sequences were manually trimmed off the reads and BAM files containing all four loci were sorted into separate BAM files for each locus of each sample.

Since ancient DNA is generally of low quality, we used a rigorous step-by-step approach to score alleles for each sample: 1) We extracted one read for each cluster of identical reads that constituted at least 5% of the reads; 2) We aligned putative alleles from both replicates; 3) We discarded all singletons differing only by C/T and G/A changes, unless an identical sequence was found in any other of the samples or among the elephant MHC alleles.

### Estimates of genetic diversity

For the diversity analyses we included three loci – exon 2, intron 2a and exon 4. Intron 2b was omitted because only half as many samples were successfully amplified and sequenced as for the other loci. We calculated genetic diversity of the mainland and Wrangel populations as observed and expected heterozygosity (H_O_ and H_E_), the number of alleles (N_A_) and the number of unique alleles per population in Arlequin 3.5.2.1^45^. We assessed the change in the frequency of heterozygous individuals between the mainland and Wrangel populations using a χ^2^ 2 × 2 contingency table, where groups were defined as the mainland and Wrangel populations and categories were defined as the number of homozygotes and heterozygotes. Allelic richness was calculated with rarefaction as implemented in adze 1.0^46^ using maximum standardized sample size of 24, equivalent to 12 diploid samples. To compare allelic richness between mammoths and African savannah elephants, we used the counts of individuals carrying an allele (see Supplementary Information). We did not include exon 4 because some of the elephant alleles were missing this part of the DQA sequence. We also created temporal statistical parsimony networks using the R-script TempNet 1.8^47^ to illustrate the change in allelic composition between the two time periods.

### Scans of selection

We scanned for signals of selection in MEGA 6^48^, and calculated the ratios of non-synonymous and synonymous mutations (*d*_N_ and *d*_S_) using the Nei-Gojobori/Jukes-Cantor method^49^. Values of *d*_N_*/d*_S_ above 1 are considered as evidence of positive selection, while values below 1 indicate purifying selection. We also performed codon-based Z-tests of selection to estimate the probabilities of positive, neutral and purifying selection. We tested selection on entire fragments of exon 2 and exon 4. Moreover, since exon 2 contains antigen-binding regions (ABR) we also analysed exon 2 split into ABR and non-ABR codons. ABR were selected according to elephant ABR^35^.

### Coalescent simulations of genetic drift

We used coalescent simulations to explore if the number of observed alleles in the Wrangel Island population could be explained under a null-hypothesis of genetic drift. To do this, we assumed that the Wrangel population can be modeled as continuous to an ancestral population having suffered a severe bottleneck ~12,000 years ago^29^. We performed coalescent simulations for a wide range of effective population sizes (N_E_B: 2–10,000 individuals) for a bottleneck of five generations of duration and a constant population size for the Wrangel population (N_E_W: 2–10,000 individuals) until extinction, both on an equally spaced log-scale. For each combination of parameters we performed 1,000 simulations for three DNA fragments of lengths 99, 89 and 92 base pairs representing the three MHC loci analyzed. In each simulation, we sampled individuals at the same time as the mean calibrated age of our samples (Table 1) and calculated the number of alleles in the ancestral population (samples > 12,000 years old) and the Wrangel Island population (<12,000 years old). The probability of observing at least 63% of the alleles of the ancestral population in the simulated Wrangel samples, corresponding to 12 out of the 19 alleles observed in our three loci, was reported. Generation time was fixed to 31 years^50^. Because new mutations in the 6,000 years between the bottleneck and the extinction of the Wrangel Island population are extremely unlikely, mutation rate and ancestral population sizes were adjusted to 1 × 10^−7^ and 100,000, respectively, in order to obtain between 13 and 25 alleles in the simulated ancestral samples. No transition bias was assumed. Simulations were performed using *fastsimcoal2*^51^ and the number of alleles estimated with *arlsumstats*, both controlled by custom R scripts^52^.

## Results

### Loss of genetic diversity

In total, we observed seven alleles in exon 2 and intron 2a and five alleles in exon 4 (see Supplementary Figures S1–S4). In each of the three loci, we observed fewer alleles in the Wrangel Island population (<10 cal ka) than in the ancestral Pleistocene population (>13 cal ka; Table 2). In general, the allele frequencies were equally distributed with most of the values ranging from 0.021 to 0.292 (see Supplementary Table S2). The mean number of alleles in the Wrangel population was 37% lower than in the mainland population.

**Figure 2:**
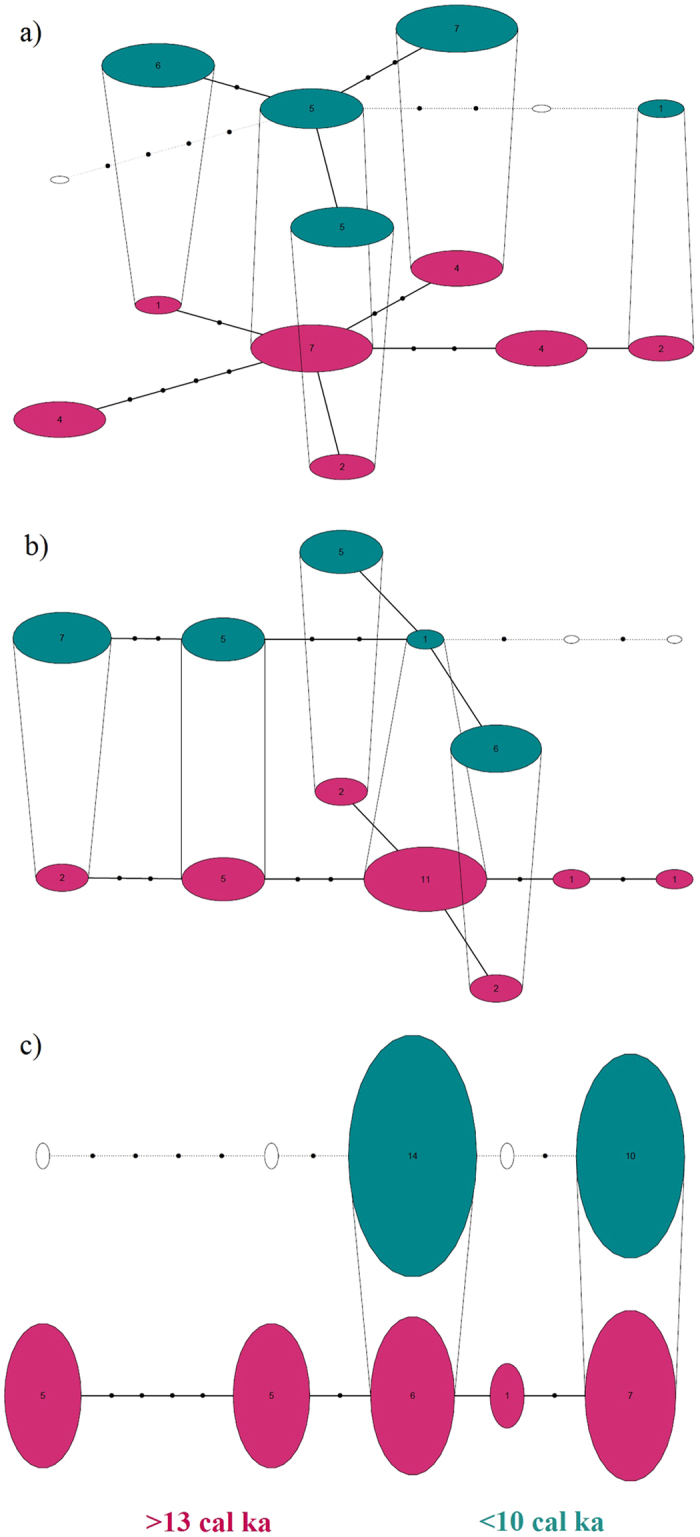
Parsimony networks of MHC DQA alleles. (**a**) exon 2, (**b**) intron 2a and (**c**) exon 4. Layers represent separate time periods before (>13 cal ka; in magenta) and after the Wrangel Island isolation (<10 cal ka; in turquoise). Numbers inside the circles indicate number of samples with the allele. Empty circles represent alleles absent in the time period.

**Figure 3:**
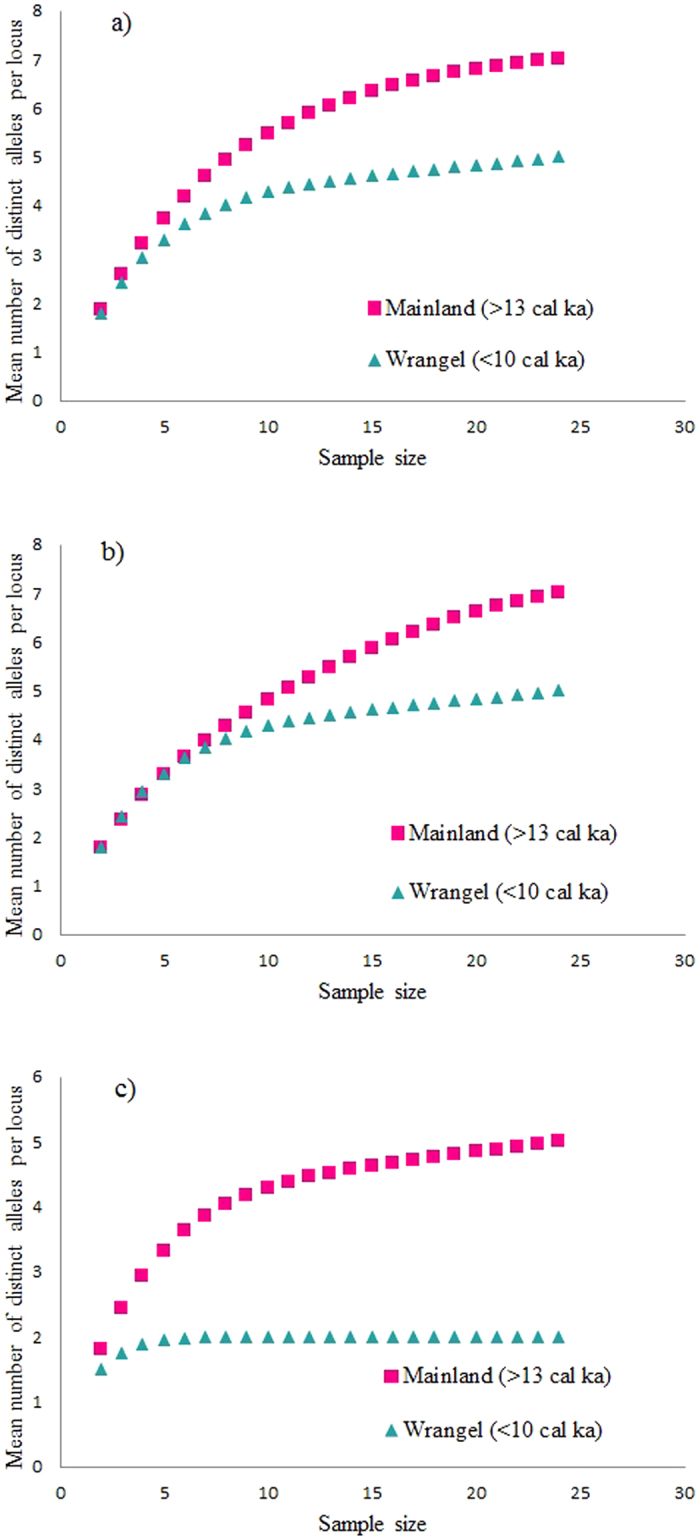
Mean number of distinct alleles per locus against number of rarefied samples. (**a**) exon 2, (**b**) intron 2a and (**c**) exon 4. Coloured shapes represent the two populations: mainland >13 cal ka (magenta squares) and Wrangel <10 cal ka (turquoise triangles).

As illustrated in the temporal statistical parsimony networks, the less frequent alleles appear to have been lost after the isolation in intron 2a and exon 4 (Fig. 2). However, we did not observe this pattern in exon 2. In all three loci, the frequency of heterozygous individuals was lower in the more recent time period (<10 cal ka). This decrease was only statistically significant in exon 4 (χ^2^ = 12, P = 0.001) and marginally significant in intron 2a (χ^2^ = 3.556, P = 0.059), whereas it was non-significant in exon 2 (χ^2^ = 1.815, P = 0.178). Plots of the mean number of distinct alleles per locus against sample size (Fig. 3) showed that for all three loci the allelic richness is higher in the mainland population compared to the population on Wrangel Island. In the comparison of allelic richness between the woolly mammoth and African savannah elephant, elephants had lower allelic richness than mammoths, even compared to the bottlenecked Wrangel population (Fig. S5).

### Positive and purifying selection

**Figure 4:**
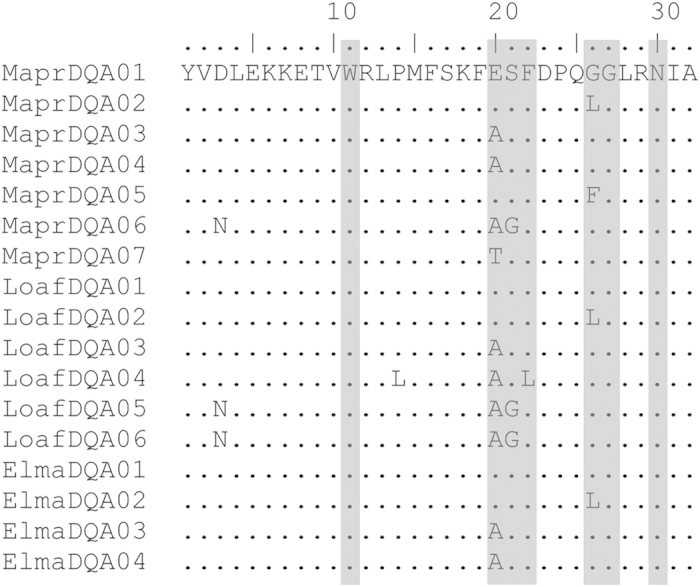
Alignment of predicted amino acid sequences of MHC DQA exon 2 in the woolly mammoth (Mapr), and African savannah (Loaf) and (Elma) Asian elephants. Highlighted are positions predicted as antigen-binding regions (ABR) in the elephant MHC^35^.

We estimated the ratio of non-synonymous to synonymous substitutions (*d*_N_/*d*_S_) in exons 2 and 4. Since exon 2 contains ABR, we performed analyses on ABR and non-ABR codons separately (Fig. 4). Although the *d*_N_*/d*_S_ ratio for ABR in exon 2 was above 1, indicating positive selection, the Z-tests of selection showed no significant deviations from neutrality (Tables 3 and 4).

The non-ABR codons of exon 2 and the entire exon 4 had *d*_N_*/d*_S_ ratios close to zero, suggesting that they are under purifying selection (Table 3). However, the Z-tests of selection were only statistically significant for exon 4 in mammoths >13 cal ka, which proved to be different from neutrality and under purifying selection. In mammoths from Wrangel Island after its isolation (<10 cal ka), none of the exons differed significantly from neutrality.

### Simulations

**Figure 5:**
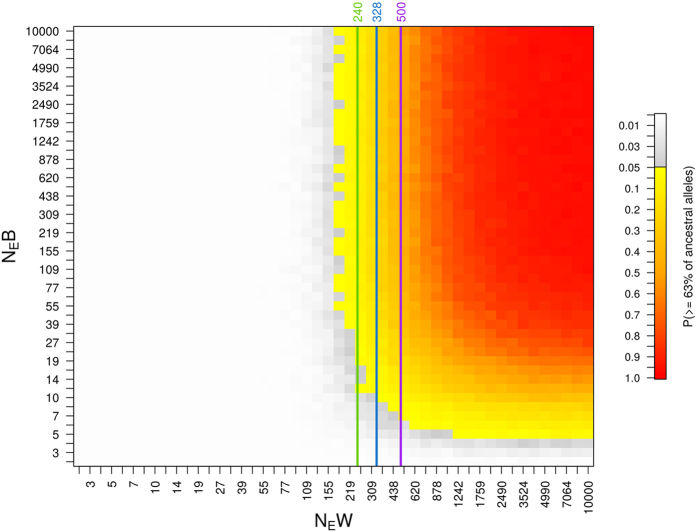
Probabilities of genetic drift being responsible for the number of alleles observed in the Wrangel Island samples as estimated by coalescent simulations. Two parameters were explored: effective population size during the bottleneck (N_E_B) and the size of the Wrangel population until extinction (N_E_W). The probability of observing at least the 63% of the alleles present in the ancestral population in the Wrangel Island samples is depicted in a yellow to red color scale. White to grey colors indicate combinations of parameters for which the simulations suggest a departure from the null-hypothesis (P < 0.05). The green^68^, blue^29^ and purple^30^ lines correspond to three estimates of the mean effective population size for Wrangel mammoths.

Our coalescent simulations suggest that the null-hypothesis of genetic drift being responsible for a reduction of more than 37% of alleles in Wrangel Island samples with respect to the ones observed in the ancestral population could be rejected only for very small effective population sizes (roughly 2-155, Fig. 5). The size of the population at the bottleneck had almost no effect on this probability as long as at least 10 individuals composed the remaining population. For scenarios in which less than four individuals survived the bottleneck, observing at least 63% of the alleles in the Wrangel samples was highly unlikely, irrespective of the effective population size after the bottleneck. Coalescent simulations performed with one and ten generations of bottleneck duration were consistent with the results presented above (see Supplementary Information and Figs S6 and S7).

## Discussion

Our results show that diversity in the MHC DQA gene decreased seemingly as a consequence of the decline in woolly mammoth population size that took place at the end of the last glaciation, when mammoths became isolated on Wrangel Island. Furthermore, the results from our coalescent simulations indicate that the observed loss of allelic diversity is consistent with what would be expected from genetic drift, and that there thus is no evidence for balancing selection having maintained diversity during the last 6,000 years of the mammoth’s existence. While it is important to remember that our analyses are based on only one gene in the MHC, our findings support the hypothesis that in small fragmented populations the stronger effect of genetic drift overshadows the power of balancing selection to maintain high MHC polymorphism. We observed a loss in allele numbers as well as heterozygosity, likely due to that the small population size on Wrangel Island led to genetic drift and inbreeding.

Although the difference in time spans of these populations should be considered, the mainland population covering ~40 ka and Wrangel population covering ~4 ka, it is unlikely that this could explain the difference in genetic diversity between these populations. Our findings of trans-species polymorphism among the woolly mammoth and extant elephants indicates that the variability in the woolly mammoth’s MHC is highly conserved. Trans-species polymorphism is a phenomenon typical for MHC when species share identical pairs of alleles as a result of strong long-term balancing selection operating on the locus^53^. There is evidence of alleles shared between mice and rats that diverged about 10 million years ago^54^ (Mya) and humans and chimpanzees with divergence dating back about 6 Mya^55^,^56^. The ancestors of the woolly mammoth split from the last common ancestor with African savannah elephant about 6.6–8.8 Mya and the origin of the clade of woolly mammoths and Asian elephant is estimated at 5.8–7.8 Mya^57^. All alleles that we found in the woolly mammoth MHC DQA are either identical to African savannah and Asian elephant alleles, or they differ in one or two positions. Thus, it is highly unlikely that mutations would have occurred during a time span of 50,000 years, and that comparing samples from a ~40 ka time period with those from a ~4 ka time period would have led to any biases in the diversity estimates.

Analyses of neutral microsatellite diversity in a previous study^29^ revealed a 30% decrease in observed heterozygosity from 0.67 ± 0.22 to 0.46 ± 0.20 comparing mammoths before and after the isolation on Wrangel Island. The mean observed heterozygosity in the MHC DQA locus dropped by 45% from 0.92 ± 0.08 to 0.50 ± 0.14. This might suggest that the positively selected MHC diversity decreased more than neutral diversity, which is consistent with empirical studies^23^,^25^ and simulation models^58^ that suggest a greater loss of variation in the MHC compared to neutral markers. However, analysis of longer or multiple loci of the MHC is needed to verify this finding.

Our comparison of genetic diversity in the woolly mammoth and African savannah elephant, measured as allelic richness, revealed that, when standardized over sample size, mammoths had more alleles on MHC DQA than elephants. At first glance, this might seem surprising given that diseases are generally more common in the tropics^59^,^60^. On the other hand, climatic and environmental fluctuations have likely been more severe in the Arctic compared to tropics, and this could potentially have led to variation in selection on MHC. We also note that the higher diversity in mainland mammoths compared to African savannah elephants could be a result of past introgression between Siberian woolly mammoths and other mammoth populations, for example during periods of gene flow from North America as identified in earlier studies^61^,^62^,^63^.

A “hyper-disease” introduced to native Pleistocene fauna by newly arrived human populations has also been suggested as an explanation of the megafauna extinction^64^,^65^. However, the lack of evidence for a major temporal shift in DQA allele frequencies, as well as the observation of higher DQA diversity in mammoths compared to African savannah elephants, do not provide any support for the hypothesis that a hyper-disease led to the extinction of the woolly mammoth and other megafaunal species.

MHC is generally considered to be a locus under balancing selection. Although we found no statistically significant support for balancing selection in the ratio of nonsynonymous to synonymous mutations, we observed other patterns that could be explained as the effects of balancing selection. We found intermediate allele frequencies close to the frequency equilibrium, as is expected when selection keeps many variants in a population, and we also found a strong trans-species polymorphism.

Hughes and Nei^66^ pointed out distinct patterns of selection on MHC in humans and mice. While they found ABR sites of MHC to be regulated by positive selection, other regions of the sequences were under purifying selection. This observation is consistent with the assumption that purifying selection maintains the general structure of the protein conserved among different mammalian taxa^67^. Accordingly, our analyses suggest that exon 4 in the woolly mammoth MHC DQA locus was under purifying selection in the mainland population before the isolation on Wrangel Island (>13 cal ka), corresponding to the pattern in human and mouse MHC. Purifying selection could also explain the generally lower number of alleles that we observed at exon 4 compared to the other two loci.

Whether the observed loss in MHC allelic diversity can be explained by genetic drift alone depends on the effective population size after mammoths became isolated on Wrangel Island. There are three previous estimates of this post-isolation effective population size. In the first^68^, the authors used the island’s size, the estimated mammoth body size, and population density of herbivores in arctic environments to estimate a mean effective size of 240 individuals in Wrangel Island, assuming a 1:1 sex ratio and a relationship of 0.5 between carrying capacity and effective population size. Second, a study based on coalescent simulations of microsatellite data indicated an effective Holocene population size of 500 individuals^30^. Recently, a third study^29^ based on a newly calibrated mutation rate obtained from two complete mammoth genomes estimated the mean effective population size on Wrangel Island to 328 individuals. All these estimates fall within the ranges for which our coalescent simulations indicate that the temporal change in the number of observed alleles on Wrangel Island can be explained by genetic drift alone. Therefore, we find no evidence that other evolutionary forces, such as balancing selection, are required to explain the maintenance of diversity at this MHC locus during the Holocene.

## Conclusions

Analyses of three short fragments of the MHC DQA gene in the woolly mammoth before and after the isolation on Wrangel Island showed that genetic diversity decreased in the Wrangel population, possibly as a result of genetic drift caused by the bottleneck and ensuing small population size. While it is important to remember that our analyses are based on only three short parts of one gene in the MHC, our results suggest that genetic drift had an effect on adaptive genetic variation in the last surviving woolly mammoth population.

Although we found no evidence of balancing selection during the Holocene, the trans-species polymorphism observed between the woolly mammoth and African savannah and Asian elephants can be considered strong evidence of historical long-term balancing selection. Our results represent the first analysis of MHC variation in an extinct species, which can be a useful primer for the study of endangered populations since MHC diversity plays a major role in immune defense and thus population persistence.

## Additional Information

**Accession codes:** KX090927-KX090945.

**How to cite this article**: Pečnerová, P. *et al*. Changes in variation at the MHC class II DQA locus during the final demise of the woolly mammoth. *Sci. Rep.* **6**, 25274; doi: 10.1038/srep25274 (2016).

## Supplementary Material

Supplementary Information

## Figures and Tables

**Table 1:** Radiocarbon dated specimens that yielded sequences for all three loci analysed in this study.

Sample	^14^C laboratory no.	^14^C age (yr BP)	^14^C age (cal yr BP)	Material	Region
L267	LU-5179	≥49 000	≥49 000	Tusk	Chukotka
L272	LU-5159	≥47 400	≥47 400	Tusk	Chukotka
L155	OxA-20038 (P23355)	47 700 ± 750	47 772	Tooth	Chukotka
L156	OxA-20039 (P23356)	46 350 ± 650	49 466	Femur	Chukotka
M2	Ua-13365	>38 000	>38 000	Tooth	Wrangel*
L163	OxA-20047 (P23363)	26 180 ± 110	30 525	Tusk	Chukotka
L152	OxA-20042 (P23359)	19 850 ± 90	23 891	Tooth	Chukotka
M6	LU-2807	20 000 ± 110	24 063	Tooth	Wrangel*
M7	LU-3510	18 030 ± 130	21 846	Bone	Wrangel*
L164	OxA-20048 (P23364)	13 935 ± 50	16 901	Tusk	Chukotka
L158	OxA-20046 (P23362)	12 385 ± 45	14 431	Humerus	Chukotka
M9	LU-2823	12 010 ± 110	13 872	Tooth	Wrangel*
M17	Ua-13372	7510 ± 80	8 318	Tooth	Wrangel Island
M23	LU-4449	6560 ± 60	7 470	Tusk	Wrangel Island
M28	GIN-6988	5610 ± 40	6 380	Tusk	Wrangel Island
L385	Ua-13359	5285 ± 65	6 074	Tooth	Wrangel Island
L387	Ua-13376	4860 ± 75	5 601	Tooth	Wrangel Island
M32	Ua-13378	4475 ± 60	5 131	Tooth	Wrangel Island
E460	LU-2756	4400 ± 40	4 969	Tusk	Wrangel Island
E461	AA40667	4389 ± 46	4 959	Bone	Wrangel Island
M38	Ua-13375	4210 ± 70	4 726	Tooth	Wrangel Island
E466	GIN-6985	3920 ± 40	4 354	Tusk	Wrangel Island
M42	AA40665	3905 ± 47	4 336	Tooth	Wrangel Island
E468	LU-2741	3730 ± 40	4 079	Tusk	Wrangel Island

Samples marked with an asterisk (*) indicate samples collected on Wrangel Island and dated to the time period when Wrangel was connected to the mainland. Sample labeled M42 corresponds to the specimen with an available complete genome^30^.

**Table 2:** Genetic diversity in different parts of the DQA gene calculated as observed heterozygosity (H_O_), expected heterozygosity (H_E_), number of alleles (N_A_) and unique alleles.

Locus	>13 cal ka (n = 12)				<10 cal ka (n = 12)			
	H_O_	H_E_	N_A_	Unique alleles	H_O_	H_E_	N_A_	Unique alleles
Exon 2	0.83	0.85	7	2	0.58	0.80	5	0
Intron 2a	0.92	0.75	7	2	0.58	0.80	5	0
Exon 4	1.00	0.80	5	3	0.33	0.51	2	0
Mean ± SD	0.92 ± 0.08	0.80 ± 0.05	6.33 ± 1.16		0.50 ± 0.14	0.70 ± 0.17	4.00 ± 1.73	

**Table 3:** Ratios of non-synonymous (*d*_N_) and synonymous (*d*_S_) substitutions in different parts of the DQA gene.

Region	No. of codons	>13 cal ka (n = 12)			<10 cal ka (n = 12)		
		*d*_N_ ± SE	*d*_S_ ± SE	*d*_N_*/d*_S_	*d*_N_ ± SE	*d*_S_ ± SE	*d*_N_*/d*_S_
Exon 2	32	0.038 ± 0.021	0.032 ± 0.021	1.19	0.028 ± 0.016	0.029 ± 0.022	0.97
Ex2 ABR	7	0.172 ± 0.116	0.070 ± 0.089	2.46	0.105 ± 0.069	0.052 ± 0.066	2.02
Ex2 Non-ABR	25	0.005 ± 0.005	0.022 ± 0.019	0.23	0.008 ± 0.008	0.023 ± 0.023	0.35
Exon 4	30	0.014 ± 0.010	0.093 ± 0.038	0.15	0 ± 0	0.061 ± 0.038	0

**Table 4:** Results from the codon-based Z-tests of positive selection, neutrality and purifying selection.

Region	No. of codons	>13 cal ka (n = 12)						<10 cal ka (n = 12)					
		Positive		Neutral		Purifying		Positive		Neutral		Purifying	
		Z	P	Z	P	Z	P	Z	P	Z	P	Z	P
Exon 2	32	0.251	0.401	0.254	0.800	0.243	1	−0.062	1	−0.065	0.949	0.063	0.475
Ex2 ABR	7	0.934	0.176	0.978	0.330	0.917	1	0.739	0.231	0.735	0.464	−0.690	1
Ex2 Non-ABR	25	−0.811	1	−0.832	0.407	0.795	0.214	0.624	1	−0.643	0.521	0.632	0.264
Exon 4	30	−2.050	1	−2.021	0.046*	1.997	0.024*	−1.575	1	−1.659	0.1	1.648	0.051

Asterisks indicate statistically significant values.
