# Supplementary material for: Changes in variation at the MHC class II DQA locus during the final demise of the woolly mammoth
Source: Sci Rep. 2016 May 4;6:25274. doi: 10.1038/srep25274 (PMC4855147; doi:10.1038/srep25274)
Supplement: Supplementary Information [file srep25274-s1.pdf]

Changes in variation at the MHC class II DQA locus during the final demise of the woolly mammoth

Patrícia Pečnerová, David Díez-del-Molino, Sergey Vartanyan, Love Dalén

Comparison of allelic richness between the woolly mammoth and African savannah elephant

To compare allelic richness between mammoths and African savannah elephants, we performed an individual-based rarefaction using the R-package[1]. Since we had no information on individual elephant genotypes and only knew the numbers of elephants carrying each elephant MHC DQA allele[2], we calculated the same measure in mammoths and used the counts of individuals carrying an allele as input for the allelic richness mammoth-elephant comparison. We corrected the number of elephant alleles according to the allelic composition at locations corresponding to our exon 2 and intron 2a (e.g. alleles LoafDQA*05 and LoafDQA*06 are identical on the particular part of exon 2 so we counted all elephants carrying these two alleles together). For these analyses, we did not include exon 4 because some of the elephant alleles were missing this part of the DQA sequence.

We found out thatAfrican savannah elephants had lower allelic richness than mammoths, even compared to the bottlenecked Wrangel population (Fig. S5).

Our comparison of genetic diversity in the woolly mammoth and African elephant, measured as allelic richness, revealed that, when standardized over sample size, mammoths had more alleles on MHC DQA than elephants. This finding is surprising because Arctic species have generally lower pathogen and parasite load than tropical species[3,4], and are hence expected to have less variability in the MHC. In an analysis of elephant MHC[2], a single allele (LoafDQA*01) had a very high frequency above 0.5 and it was suggested that this could have been caused by that allele LoafDQA*01 has provided a selective advantage against a recent prevalent disease. Directional selection favoring a specific allele could thus be a possible explanation to why the MHC diversity is lower in elephants compared to woolly mammoths.

Supplementary information

Fig. S1

The woolly mammoth MHC DQA alleles of exon 2, compared to the alleles found in the African and Asian elephants.

Fig. S2

The woolly mammoth MHC DQA alleles of intron 2a, compared to the alleles found in the African and Asian elephants.

Fig. S3

The woolly mammoth MHC DQA alleles of exon 4, compared to the alleles found in the African and Asian elephants.

Fig. S4

The translated sequences of the woolly mammoth MHC DQA alleles of exon 2, compared to the alleles found in the African and Asian elephants.

Fig. S5

A comparison of allelic richness between the woolly mammoth and African elephant.

a) exon 2 and b) intron 2a of the MHC DQA gene, estimated from the number of individuals carrying an allele. Coloured shapes represent populations: mainland mammoths >13 cal ka (magenta squares), Wrangel mammoths <10 cal ka (turquoise triangles) and African elephants (orange diamonds).

Fig. S6

Probabilities of genetic drift being responsible for the number of alleles observed in the Wrangel Island samples as estimated by coalescent simulations for a bottleneck of a single generation of duration. Two parameters were explored: effective population size during the bottleneck (N_EB) and the size of the Wrangel population until extinction (N_EW).

The probability of observing at least the 63% of the alleles present in the ancestral population in the Wrangel Island samples is depicted in a yellow to red color scale. White to grey colors indicate combinations of parameters for which the simulations suggest a departure from the null-hypothesis ($P < 0.05$). The green[5], blue[6] and purple[7] lines correspond to three estimates of the mean effective population size for Wrangle mammoths.

Fig. S7

Probabilities of genetic drift being responsible for the number of alleles observed in the Wrangel Island samples as estimated by coalescent simulations for a bottleneck of ten generations of duration. Two parameters were explored: effective population size during the bottleneck (N_EB) and the size of the Wrangel population until extinction (N_EW). The probability of observing at least the 63% of the alleles present in the ancestral population in the Wrangel Island samples is depicted in a yellow to red color scale. White to grey colors indicate combinations of parameters for which the simulations suggest a departure from the null-hypothesis ($P < 0.05$). The green[5], blue[6] and purple[7] lines correspond to three estimates of the mean effective population size for Wrangle mammoths.

Table S1

Information about primer sequences used for amplification and sequencing of the four fragments of MHC DQA in the woolly mammoth.

Table S2

Allele frequencies at the three loci in the mainland population (>13 cal ka) and in the Wrangel Island population (<10 cal ka).

Fig.S1

```
                    10        20        30        40        50        60        70        80        90
            ....|....|....|....|....|....|....|....|....|....|....|....|....|....|....|....|....|....|....|....
MaprDQA01   TCTACGTGGACCTGGAGAAGAAGGAGACTGTGTGGCGACTACCTATGTTTAGCAAATTTGAAAGTTTTGACCCACAAGGTGGACTGAGGAACATAGCTG
MaprDQA02   ........................................G....................................CT....................
MaprDQA03   ........................................G...................C......................................
MaprDQA04   .G......................................G........C..........C.TCG..................................
MaprDQA05   ........................................G....................................TT....................
MaprDQA06   ........A...............................G...................C.G.A..................................
MaprDQA07   ........................................G..................AC......................................
LoafDQA01   ...................................................................................................
LoafDQA02   ...................................A....G....................................CT....................
LoafDQA03   ........................................G...................C......................................
LoafDQA04   .G......................................G.T......C..........C.TCGC.................................
LoafDQA05   ........A...............................G...................C.G.A..................................
LoafDQA06   ........A...............................G...................C.G.A..................................
ElmaDQA01   ...................................................................................................
ElmaDQA02   ........................................G....................................CT....................
ElmaDQA03   ........................................G...................C......................................
ElmaDQA04   .G......................................G........C..........C.TCG..................................
```

Fig.S2

```
                      10        20        30        40        50        60        70        80
              ....|....|....|....|....|....|....|....|....|....|....|....|....|....|....|....|....|....
MaprDQA01    ATACCAAGTCTCACTTCATTCTTCCTTAGAGATAGATACCCATCTCACCATGCTATAGAACTCTCTCCTTTACAAGGAGTTCCCACATC
MaprDQA02    ............................................C.....CT.....................................
MaprDQA03    ................T...........................C.....CT.....................................
MaprDQA04    .........................A..................C.....CT.....................................
MaprDQA05    ..................................................CT..G..................................
MaprDQA06    .........................A..................C...........................................G
MaprDQA07    ................T.................................CT..G..............................T...
LoafDQA01    .C....................................................................................T..
LoafDQA02    ............................................C.....CT.....................................
LoafDQA03    ................T.................................CT..G..............................T...
LoafDQA04    ............................................C.....CT.....................................
LoafDQA05    .........................A........................CT..G..................................
LoafDQA06    .........................A..................C.....CT....................................G
ElmaDQA01    .........................................................................................
ElmaDQA02    ............................................C.....CT.....................................
ElmaDQA03    ................T.................................CT..G..............................T...
ElmaDQA04    ............................................C.....CT..................................G..
```

Fig.S3

```
                   10        20        30        40        50        60        70        80        90
           ....|....|....|....|....|....|....|....|....|....|....|....|....|....|....|....|....|....|..
MaprDQA01  GACAGAGCTGACTGAGACTGTGGTCTGTGCCTTGGGATTGGTCGTGGGCCTCGTGGGCATCGTGGTGGGCACCATCCTCATCATCCGAGGCC
MaprDQA02  C....................A..................................................A..G..........A.....
MaprDQA03  ....................................G..............T.......................T................
MaprDQA04  .................................................................................T....A.....
MaprDQA05  ...................................................T........................................
LoafDQA01  ............................................................................................
LoafDQA02  C....................A..................................................A..G..........A.....
ElmaDQA01  ............................................................................................
ElmaDQA02  C....................A..................................................A..G..........A.....
ElmaDQA03  ....................................G..............T.......................T................
```

Fig.S4

```
                         10        20        30
               ....|....|....|....|....|....|
MaprDQA01      TELTETVVCALGLVVGLVGIVVGTILIIRG
MaprDQA02      ........................M...Q.
MaprDQA03      ..............................
MaprDQA04      ............................Q.
MaprDQA05      ..............................
LoafDQA01      ..............................
LoafDQA02      ........................M...Q.
ElmaDQA01      ..............................
ElmaDQA02      ........................M...Q.
ElmaDQA03      ..............................
```

Fig.S5

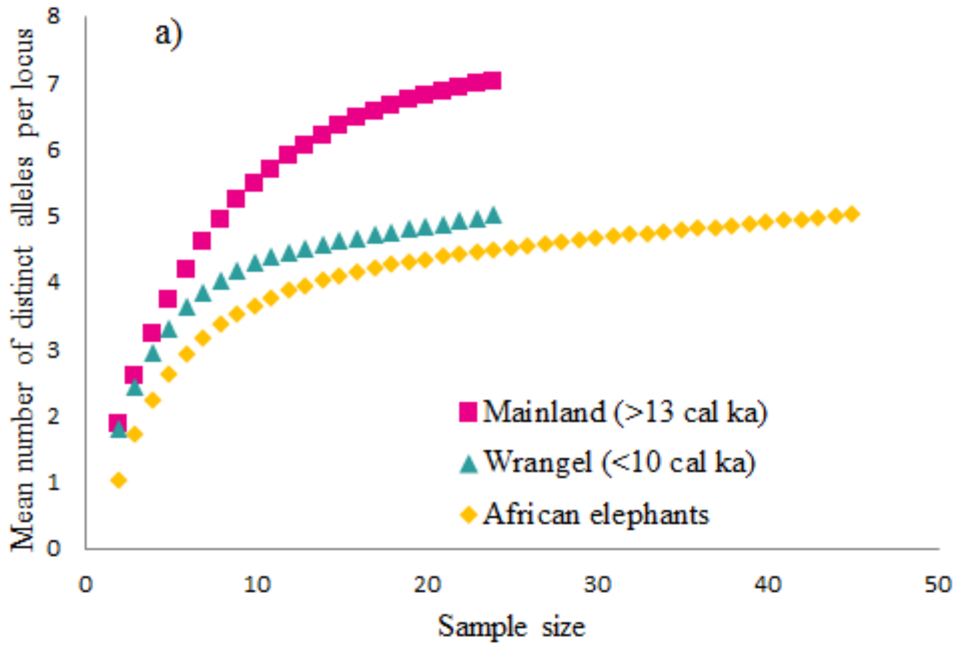
a)
Mean number of distinct alleles per locus
Sample size
Mainland (>13 cal ka)
Wrangel (<10 cal ka)
African elephants
0
1
2
3
4
5
6
7
8
0
10
20
30
40
50

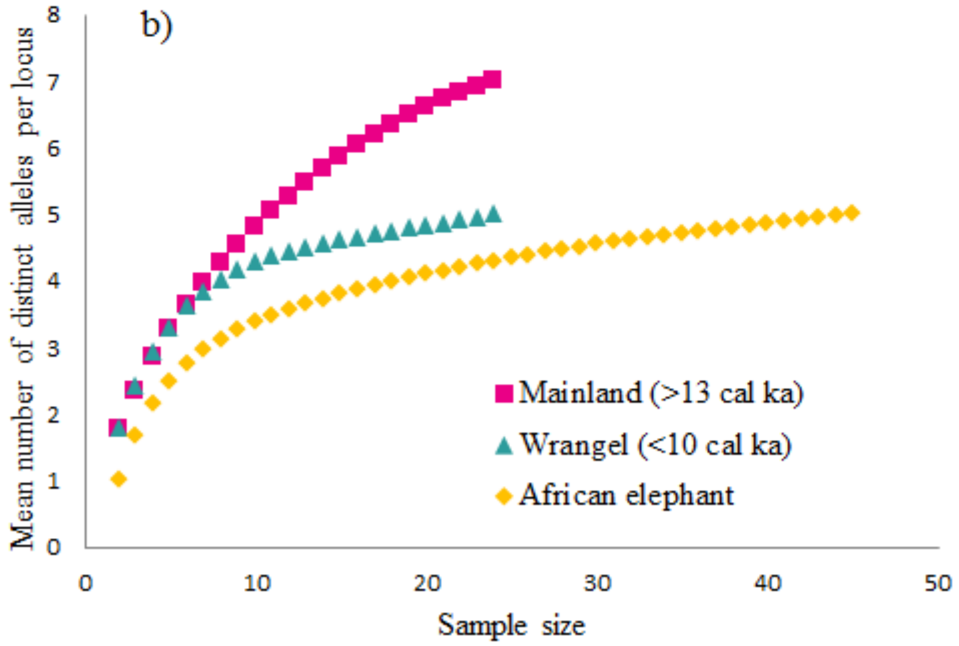
b)
Mean number of distinct alleles per locus
Sample size
Mainland (>13 cal ka)
Wrangel (<10 cal ka)
African elephant
0
1
2
3
4
5
6
7
8
0
10
20
30
40
50

Fig.S6

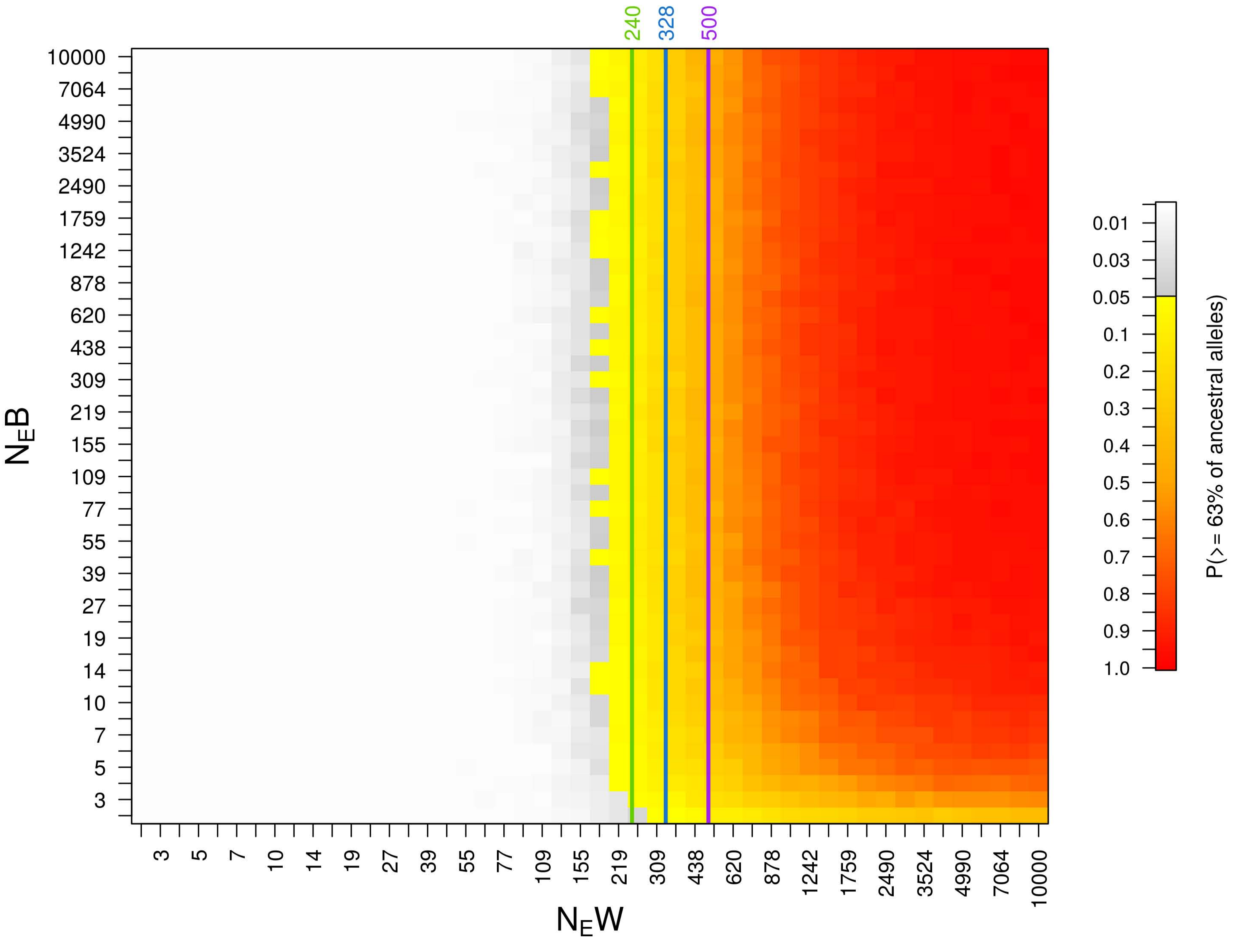

240
328
500
10000
7064
4990
3524
2490
1759
1242
878
620
438
309
219
155
109
77
55
39
27
19
14
10
7
5
3
N_EB
3
5
7
10
14
19
27
39
55
77
109
155
219
309
438
620
878
1242
1759
2490
3524
4990
7064
10000
N_EW
0.01
0.03
0.05
0.1
0.2
0.3
0.4
0.5
0.6
0.7
0.8
0.9
1.0
P(>= 63% of ancestral alleles)

Fig.S7

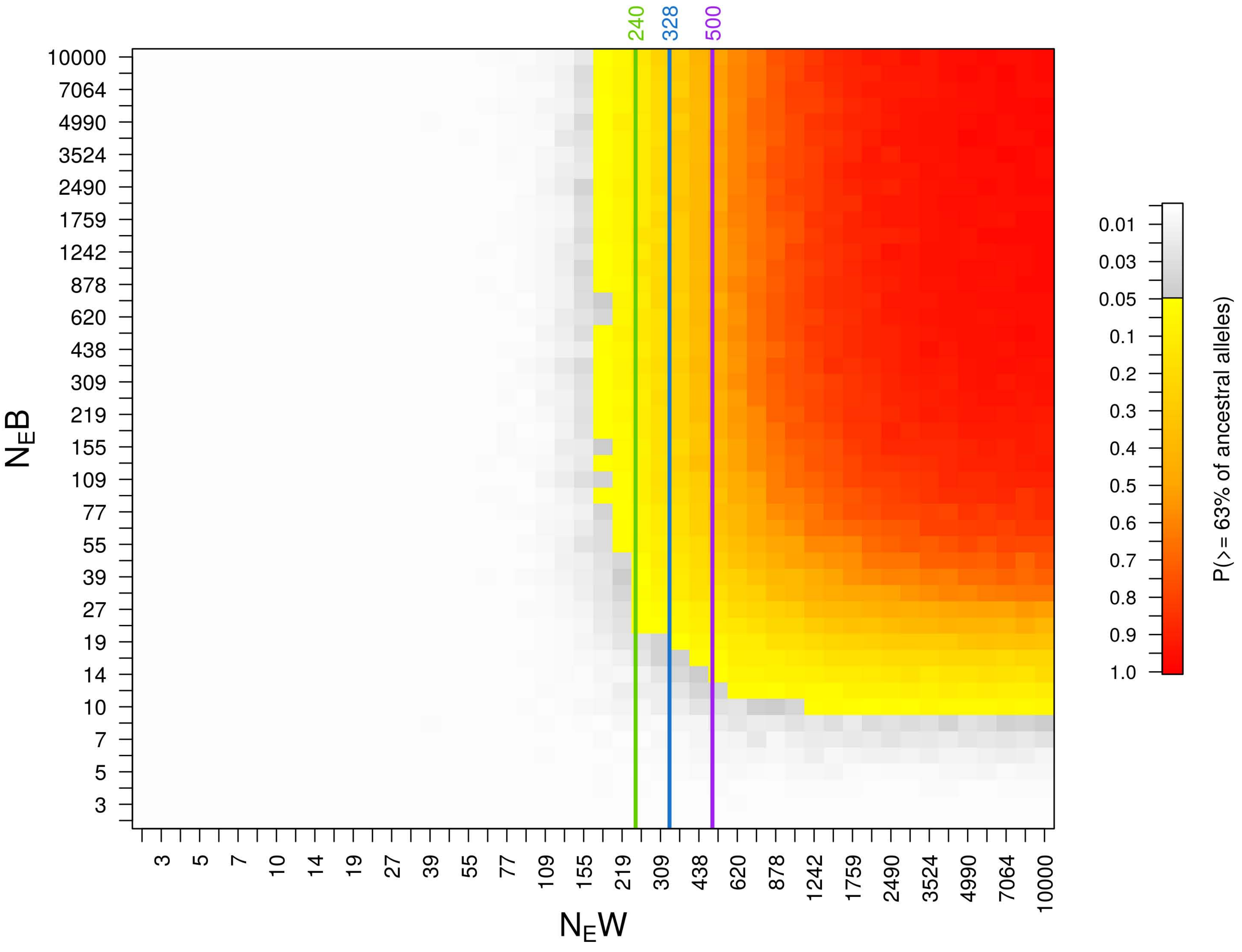

240
328
500
10000
7064
4990
3524
2490
1759
1242
878
620
438
309
219
155
109
77
55
39
27
19
14
10
7
5
3
N_EB
3
5
7
10
14
19
27
39
55
77
109
155
219
309
438
620
878
1242
1759
2490
3524
4990
7064
10000
N_EW
0.01
0.03
0.05
0.1
0.2
0.3
0.4
0.5
0.6
0.7
0.8
0.9
1.0
P(>= 63% of ancestral alleles)

Table S1. Information about primer sequences used for amplification and sequencing of the four fragments of MHC DQA1 in the woolly mammoth.

Primer name	Primer sequence (5' to 3')	T_a (°C)	Fragment length w. primers (bp)
MPDQA-1F	GAATTTGATGGAGATGAGCTGT	52	141
MPDQA-1R	GTCCAAGTTGTGTTTTAGCA		
MPDQA-2F	GTGATTCCCAAGCCAAAGG	50	151
MPDQA-2R	TGGGAAACACAGTCACTTC		
MPDQA-3F	CTCTTTATTGAATCTATCCCTCC	52	132
MPDQA-3R	TTCAGCAATTACCATGAGAA		
MPDQA-4F	CCTGAGATTCCAACCCCTAT	54	132
MPDQA-4R	GGAGACACTTCCTGAGCGTA		
MPDQA-i-1F	TGGAGATGAGCTGTTCTACGT	52	112
MPDQA-i-1R	AGCTATGTTCCTCAGTCCA		
MPDQA-i-3F	ATCTATCCCTCCATACCAAGTC	52	121

Table S2. Allele frequencies at the three loci in the mainland population (>13 cal ka) and in the Wrangel Island population (<10 cal ka).

Locus: Loc1	Mainland > 13 000 cal ka	Wrangel <10 000 cal ka	All weighted	All unweighted
Sample size	12	12	24	24
MaprDQA*01	0.042	0.250	0.146	0.146
MaprDQA*02	0.167	0.000	0.083	0.083
MaprDQA*03	0.292	0.208	0.250	0.250
MaprDQA*04	0.167	0.000	0.083	0.083
MaprDQA*05	0.083	0.042	0.063	0.063
MaprDQA*06	0.167	0.292	0.229	0.229
MaprDQA*07	0.083	0.208	0.146	0.146

Locus: Loc3	Mainland > 13 000 cal ka	Wrangel <10 000 cal ka	All weighted	All unweighted
Sample size	12	12	24	24
MaprDQA*01	0.208	0.208	0.208	0.208
MaprDQA*02	0.458	0.042	0.250	0.250
MaprDQA*03	0.083	0.208	0.146	0.146
MaprDQA*04	0.083	0.250	0.167	0.167
MaprDQA*05	0.042	0.000	0.021	0.021
MaprDQA*06	0.083	0.292	0.188	0.188
MaprDQA*07	0.042	0.000	0.021	0.021

Locus: Loc4	Mainland > 13 000 cal ka	Wrangel <10 000 cal ka	All weighted	All unweighted
Sample size	12	12	24	24
MaprDQA*01	0.250	0.583	0.417	0.417
MaprDQA*02	0.208	0.000	0.104	0.104
MaprDQA*03	0.292	0.417	0.354	0.354
MaprDQA*04	0.208	0.000	0.104	0.104
MaprDQA*05	0.042	0.000	0.021	0.021

1. Jacobs, J. *Individual Based Rarefaction using R-package*, <http://www.jennajacobs.org/R/rarefaction.html> (2011).
2. Archie, E. A. *et al.* Major histocompatibility complex variation and evolution at a single, expressed DQA locus in two genera of elephants. *Immunogenetics* **62**, 85-100, doi:DOI 10.1007/s00251-009-0413-8 (2010).
3. Kutz, S. J. *et al.* The Arctic as a model for anticipating, preventing, and mitigating climate change impacts on host-parasite interactions. *Vet Parasitol* **163**, 217-228, doi:10.1016/j.vetpar.2009.06.008 (2009).
4. Weber, D. S. *et al.* Low MHC variation in the polar bear: implications in the face of Arctic warming? *Anim Conserv* **16**, 671-683, doi:10.1111/acv.12045 (2013).
5. Nyström, V. et al. Temporal genetic change in the last remaining population of woolly mammoth. Proc R Soc B 277, 2331–2337, doi:10.1098/rspb.2010.0301 (2010).
6. Palkopoulou, E. et al. Complete Genomes Reveal Signatures of Demographic and Genetic Declines in the Woolly Mammoth. Curr Biol 25, 1395-1400, doi:10.1016/j.cub.2015.04.007 (2015).
7. Nyström, V. et al. Microsatellite genotyping reveals end-Pleistocene decline in mammoth autosomal genetic variation. Mol Ecol 21, 3391-3402, doi: 10.1111/j.1365-294X.2012.05525.x (2012).
